# Alpha-linolenic acid in cancer: a predictive fate-switch framework for tumor lipid reprogramming

**DOI:** 10.3389/fonc.2026.1928431

**Published:** 2026-08-12

**Authors:** Jinrui Zheng, Xinxin Li, Hewei Gong, Jinshan Yang, Jingxuan Liu, Yuxin Lin, Yantao Lou, Jiahui Wang, Chunhua Lin

**Affiliations:** 1 School of Clinical Medicine, Shandong Second Medical University, Weifang, Shandong, China; 2 Department of Urology, The Affiliated Yantai Yuhuangding Hospital of Qingdao University, Yantai, China

**Keywords:** alpha-linolenic acid, cancer metabolism, fatty acid desaturase, ferroptosis, lipid remodeling, nutritional stratification, predictive framework

## Abstract

Alpha-linolenic acid (ALA) is the major plant-derived omega-3 fatty acid in human diets, yet its relevance to cancer is often narrowly interpreted as a precursor to eicosapentaenoic acid and docosahexaenoic acid. Here we propose a predictive fate-switch framework that treats ALA as a dietary lipid input whose biological output is dictated by tumor-intrinsic routing after uptake. Five competing fates are identified—membrane phospholipid incorporation; elongation and desaturation mediated by fatty acid desaturases (FADSs) and elongases of very-long-chain fatty acids (ELOVLs); lipid-droplet buffering; mitochondrial β-oxidation; and peroxidation-prone phospholipid enrichment—each governed by distinct molecular checkpoints. We organize these routes into three dominant but potentially overlapping tumor metabolic states: conversion-prone, storage-buffered, and peroxidation-vulnerable, which yield specific and testable predictions. For example, ALA is expected to exert stronger oxidative or ferroptosis-related effects in tumors with high polyunsaturated fatty acid (PUFA)-phospholipid incorporation and weak peroxide defense, but weaker effects where it is sequestered into neutral lipid pools. Accordingly, future studies should prioritize direct assessment of ALA fate through lipidomics, isotope tracing, and redox profiling rather than relying on intake or exposure alone. We recognize that direct ALA-specific evidence remains uneven across mechanisms—most notably in ferroptosis. Rather than undermining the framework, this unevenness defines its priority testing ground, transforming evidence gaps into actionable explorations.

## 1 Introduction

Metabolic reprogramming is a defining feature of malignant disease. Although altered glucose metabolism has dominated much of cancer-metabolism research, lipid metabolism is now recognized as an equally important determinant of tumor growth, stress adaptation, membrane dynamics, redox balance, immune interaction, and therapeutic response (1, 2). Tumor cells frequently increase fatty-acid uptake, *de novo* lipogenesis, acyl-CoA activation, phospholipid remodeling, lipid-droplet storage, and fatty acid β-oxidation (FAO). These adaptations do not merely provide fuel or biomass; they influence how malignant cells organize membranes, control signaling platforms, tolerate oxidative stress, and respond to nutrient fluctuation (1–3).

Within this lipid-centered view of cancer, dietary fatty acids are more than background nutritional variables. They are exogenous substrates that can be selectively taken up and rerouted by tumor cells. Omega-3 polyunsaturated fatty acids (PUFAs) have long been studied in relation to inflammation, membrane organization, cachexia, and treatment response, but most attention has focused on marine-derived long-chain omega-3 PUFAs, especially eicosapentaenoic acid (EPA) and docosahexaenoic acid (DHA) (4, 5). By contrast, alpha-linolenic acid (ALA; 18:3n-3), the essential plant-derived omega-3 fatty acid, is often treated mainly as an upstream precursor of EPA and DHA (6–8). That interpretation is incomplete for cancer biology.

ALA is not simply a nutrient waiting to be converted. Once delivered to tissues, ALA can enter several competing routes: incorporation into membrane phospholipids, esterification into neutral lipids, fatty acid β-oxidation (FAO), enzymatic elongation and desaturation, or generation of oxidized lipid species (6–9). In tumors, the relative flux through these routes may be altered by fatty-acid uptake programs, omega-6 competition, acyl-CoA synthetase activity, FADS/ELOVL capacity, lipid-droplet buffering, antioxidant defense, and microenvironmental nutrient stress (10–13). Therefore, the same ALA exposure may have different biological meanings in different cancers.

This review proposes a predictive fate-switch framework for interpreting ALA in cancer. The key question is not whether ALA is universally beneficial or harmful, but how a given tumor routes ALA after uptake and which molecular determinants govern that routing decision. If ALA is incorporated into membrane phospholipids, it may alter membrane fluidity, lipid rafts, receptor clustering, and peroxidation susceptibility, as suggested by broader PUFA and phospholipid remodeling evidence (12, 13). If it is converted through the FADS/ELOVL axis, it may generate longer-chain PUFAs with distinct signaling and redox consequences (8, 12). If it is stored in lipid droplets, it may be buffered from peroxidation and become part of a tumor-adaptive lipid reserve (11, 13). If it is oxidized, it may support energy production under metabolic stress (9). Thus, ALA exposure alone is insufficient to predict outcome; intracellular fate is the biologically decisive variable.

This perspective helps explain why the ALA-cancer literature appears heterogeneous. Experimental studies often report anti-proliferative, pro-apoptotic, anti-inflammatory, or anti-migratory effects (14, 15), whereas epidemiologic and nutritional findings are more variable (16, 17). Rather than treating this inconsistency as simple contradiction, a fate-switch framework suggests that ALA effects depend on tumor lineage, molecular subtype, lipid-metabolic phenotype, redox state, and host diet. The purpose of this review is therefore not to catalogue every reported effect of ALA. Instead, it integrates biochemical ALA metabolism with cancer-associated lipid reprogramming, proposes dominant tumor metabolic states that may predict ALA responsiveness, and derives testable hypotheses for future studies. This review is primarily intended to guide the design of experimental and translational studies in cancer lipid metabolism, and may also inform stratification thinking in nutritional oncology research.

Previous reviews have mainly summarized the reported antitumor effects of ALA or discussed omega-3 polyunsaturated fatty acids as a group, often treating ALA primarily as a precursor of EPA and DHA. In contrast, the present review focuses on tumor-intrinsic routing after ALA uptake. It links five competing intracellular fates to three dominant metabolic states and proposes operational criteria through which these predictions can be directly tested. The novelty of this framework therefore lies not in assigning ALA a universal anticancer effect, but in explaining why the same ALA exposure may produce different biological outcomes across tumor metabolic contexts.

## 2 Literature search and evidence scope

**Figure 1:**
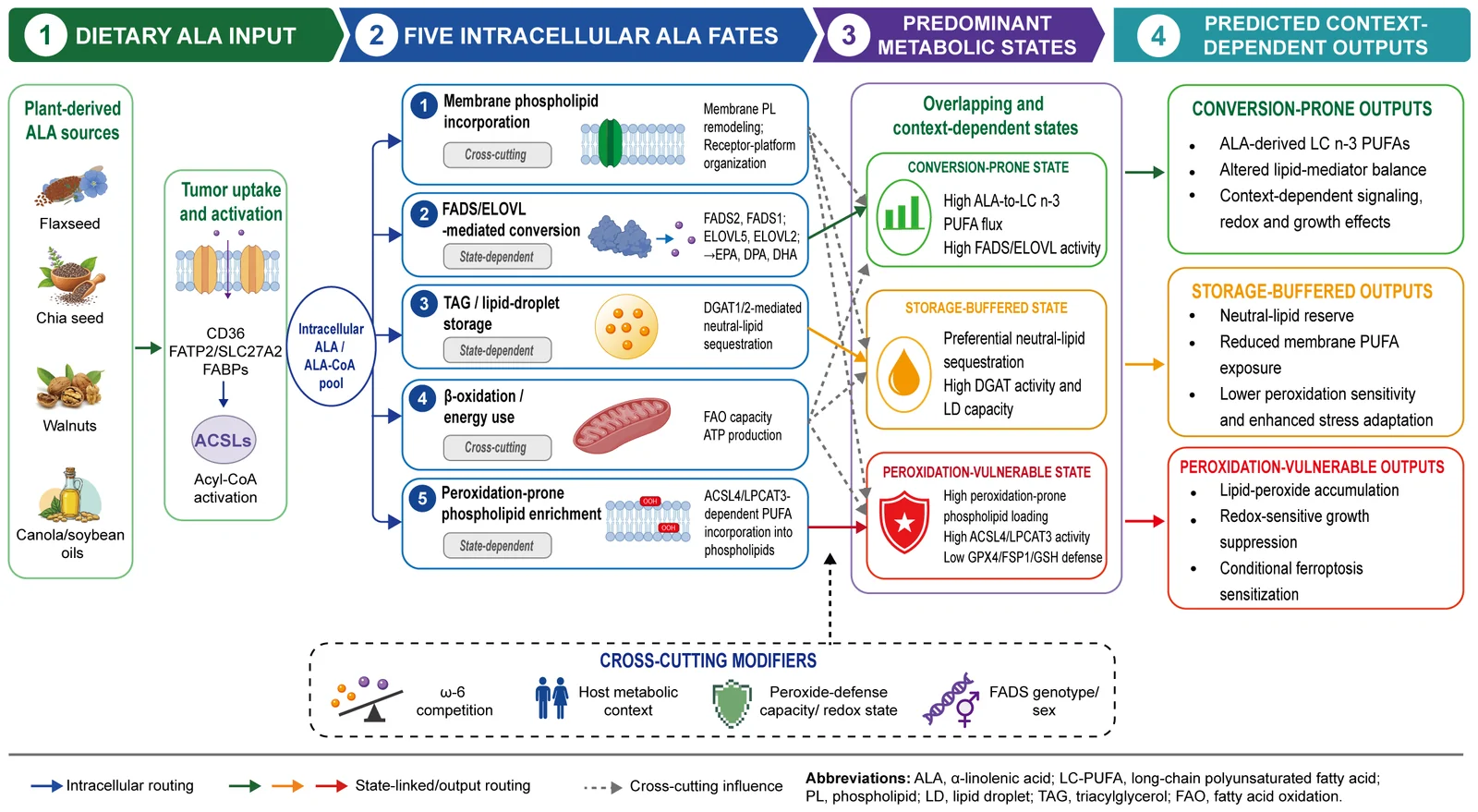
The predictive ALA fate-switch framework for tumor lipid metabolism. Dietary ALA from plant sources enters tumor cells via CD36, FATP2/SLC27A2, and FABPs, and is activated by ACSLs to form the intracellular ALA-CoA pool. Once imported, ALA is routed into five competing fates: membrane phospholipid incorporation, FADS/ELOVL-mediated conversion to long-chain n-3 PUFAs (EPA, DPA, DHA), TAG/lipid-droplet storage, β-oxidation for energy, and peroxidation-prone phospholipid enrichment. Membrane incorporation and β-oxidation function as cross-cutting routes that modify multiple phenotypes. These fates converge into three predominant but overlapping tumor metabolic states — conversion-prone, storage-buffered, and peroxidation-vulnerable — each generating distinct predicted biological outputs. Four cross-cutting modifiers (ω-6 competition, host metabolic context, peroxide-defense capacity/redox state, and FADS genotype/sex) operate across all states and shape the direction and magnitude of ALA routing. Evidence strength varies across pathways: some relationships are supported by direct ALA studies, whereas others are extrapolated from broader PUFA biology or remain hypothesis-generating, as detailed in the text and tables. ALA, α-linolenic acid; LC-PUFA, long-chain polyunsaturated fatty acid; PL, phospholipid; LD, lipid droplet; TAG, triacylglycerol; FAO, fatty acid oxidation; FADS, fatty acid desaturase; ELOVL, elongation of very long chain fatty acids protein; ACSL, acyl-CoA synthetase long-chain family member; LPCAT3, lysophosphatidylcholine acyltransferase 3; DGAT, diacylglycerol acyltransferase; GPX4, glutathione peroxidase 4; FSP1, ferroptosis suppressor protein 1; GSH, glutathione; CD36, cluster of differentiation 36; FATP2, fatty acid transport protein 2; SLC27A2, solute carrier family 27 member 2; FABP, fatty acid-binding protein; EPA, eicosapentaenoic acid; DPA, docosapentaenoic acid; DHA, docosahexaenoic acid.

This narrative review was informed by searches of PubMed and Web of Science from database inception to 30 June 2026. Search terms included combinations of “alpha-linolenic acid”, “ALA”, “cancer”, “tumor”, “lipid metabolism”, “fatty acid uptake”, “fatty acid desaturase”, “FADS”, “ELOVL”, “lipid droplet”, “fatty acid oxidation”, “phospholipid remodeling”, “ferroptosis”, “lipid peroxidation”, “tumor microenvironment”, and “polyunsaturated fatty acids”. Titles and abstracts were screened for relevance, followed by full-text assessment of potentially relevant articles. Reference lists of key original studies and reviews were also examined to identify additional publications. Priority was given to mechanistic and preclinical studies that directly examined ALA uptake, incorporation, conversion, storage, oxidation, or biological effects in cancer models. Where direct ALA-specific evidence was unavailable, studies of other PUFAs were included only to establish mechanistic plausibility, and such evidence is explicitly identified as extrapolative in the relevant sections. Because this was a narrative rather than a systematic review, no formal meta-analysis or risk-of-bias assessment was performed. The overall logic of the predictive ALA fate-switch framework is summarized in **Figure 1**.

## 3 Biochemical entry points of ALA metabolism

ALA is the parent essential fatty acid of the omega-3 series (18). Humans cannot introduce double bonds beyond the delta-9 position *de novo*, so ALA must be obtained from dietary sources such as flaxseed, chia seed, walnuts, soybean oil, and canola oil (19, 20). Nutritionally, ALA is important because it is the dominant omega-3 fatty acid consumed in many populations, particularly where marine omega-3 intake is low (6, 21). Biologically, however, its effects cannot be inferred from intake alone. Circulating ALA must first reach tissue compartments, enter cells, and be activated before it can influence membrane composition, signaling, energy metabolism, or lipid peroxidation (20).

ALA uses the general long-chain fatty-acid handling machinery. Transport-associated proteins, intracellular fatty-acid-binding proteins, and acyl-CoA synthetases cooperate to determine whether imported fatty acids are incorporated into phospholipids, stored, oxidized, or elongated (22). Fatty acid transport protein 2 (FATP2) is relevant because it can couple uptake with acyl-CoA formation and channel exogenous n-3 fatty acids toward specific phospholipid pools (23, 24). This means that uptake is not merely permissive; it is an early routing step that can bias ALA toward structural, oxidative, or signaling-related fates.

The canonical metabolic route of ALA involves conversion to longer-chain omega-3 PUFAs. FADS2 catalyzes the initial delta-6 desaturation step and functions as a rate-limiting gatekeeper, followed by elongation and desaturation reactions involving ELOVL5, FADS1, ELOVL2, and peroxisomal steps required for DHA synthesis (25, 26). FADS1/2 and ELOVL2/5 are membrane-bound enzymes located predominantly in the endoplasmic reticulum (27), whereas the terminal steps of DHA synthesis additionally involve peroxisomal β-oxidation (25, 26). This pathway is quantitatively limited in humans: conversion to EPA is modest, conversion to DHA is low, and both processes vary by sex, substrate background, and genetic variation. Consequently, ALA should not be viewed as an interchangeable substitute for EPA or DHA. A substantial fraction may remain as ALA-containing lipids, be oxidized, or be redistributed into other lipid compartments (6).

ALA metabolism is further shaped by competition with linoleic acid (LA) and other omega-6 substrates. ALA and linoleic acid share desaturases, elongases, and esterification pathways, particularly at the FADS2 entry step (28, 29). A high omega-6 dietary background can reduce ALA conversion efficiency and alter downstream PUFA profiles. In contemporary Western diets, the omega-6 to omega-3 ratio typically ranges from 15:1 to 20:1 (28, 30), a disproportion that substantially reduces ALA conversion efficiency through substrate competition at FADS2 (29). At such excess omega-6 intakes, LA outcompetes ALA for the rate-limiting delta-6 desaturation step, suppressing downstream production of EPA and DHA even when dietary ALA intake is adequate (29). This is essential for oncology: a tumor exposed to ALA in a high omega-6 environment may generate a different phospholipid and mediator profile from one exposed to ALA under lower omega-6 pressure (31, 32). Conversion efficiency is further modified by sex: women show higher rates of ALA conversion to EPA and DHA than men, an effect attributed to estrogen-mediated upregulation of FADS2 and related desaturase activity (33, 34). This sex-related difference is directly relevant to breast and gynecological cancer research and indicates that host hormonal status should be considered alongside dietary background when interpreting ALA fate. ALA biology should therefore be interpreted as a competitive lipid-routing problem shaped by omega-6 load, sex, and genetic background rather than a single-nutrient effect (26, 35).

## 4 Tumor reprogramming of five competing ALA fates program

**Figure 2:**
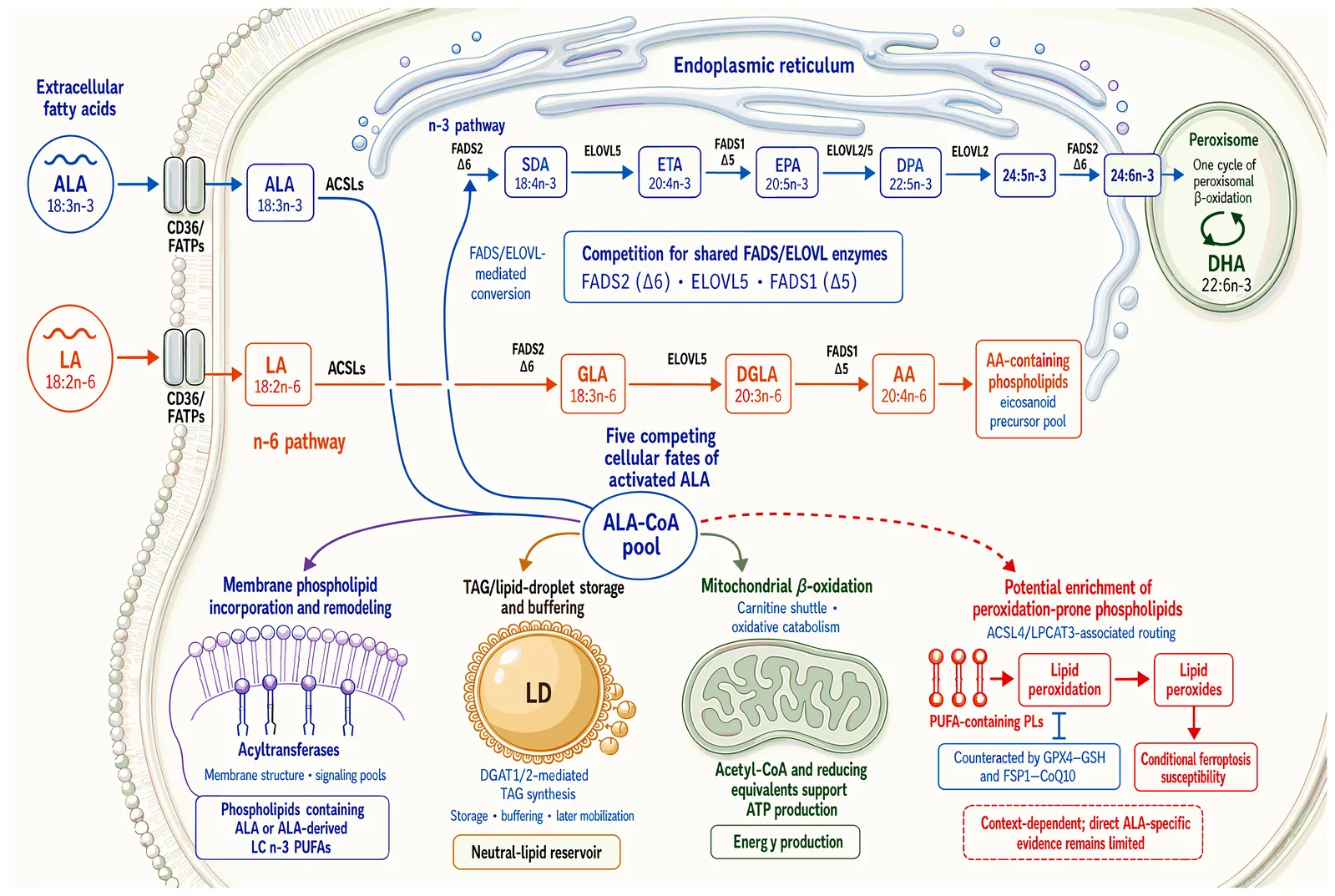
Biochemical routing of ALA through five competing cellular fates underlying three proposed metabolic states. Extracellular ALA and LA are taken up via CD36/FATPs and activated by ACSLs. The n-3 and n-6 pathways compete for shared FADS/ELOVL enzymes. After activation to ALA-CoA, ALA is partitioned among five competing routes: membrane phospholipid incorporation and remodeling, FADS/ELOVL-mediated conversion to long-chain n-3 PUFAs, TAG/lipid-droplet storage and buffering, mitochondrial β-oxidation, and enrichment of peroxidation-prone phospholipids. Their principal outputs include membrane and signaling remodeling, EPA/DPA/DHA formation, neutral-lipid buffering, ATP production, and conditional lipid peroxidation or ferroptosis susceptibility. FADS/ELOVL-mediated conversion, lipid-droplet storage, and peroxidation-prone phospholipid enrichment provide the biochemical basis for the conversion-prone, storage-buffered, and peroxidation-vulnerable states, respectively, whereas membrane remodeling and β-oxidation act as cross-cutting routes. Peroxidation or ferroptosis susceptibility depends on the balance between ACSL4/LPCAT3-mediated PUFA-phospholipid routing and GPX4–GSH/FSP1–CoQ10 antioxidant defenses; direct ALA-specific evidence for this branch remains limited. AA, arachidonic acid; ACSL, acyl-CoA synthetase long-chain family member; ALA, α-linolenic acid; ATP, adenosine triphosphate; CD36, cluster of differentiation 36; CoA, coenzyme A; CoQ10, coenzyme Q10; DGAT, diacylglycerol acyltransferase; DGLA, dihomo-γ-linolenic acid; DHA, docosahexaenoic acid; DPA, docosapentaenoic acid; ELOVL, elongation of very long-chain fatty acids protein; EPA, eicosapentaenoic acid; ETA, eicosatetraenoic acid; FADS, fatty acid desaturase; FATP, fatty acid transport protein; FSP1, ferroptosis suppressor protein 1; GLA, γ-linolenic acid; GPX4, glutathione peroxidase 4; GSH, glutathione; LA, linoleic acid; LC n-3 PUFA, long-chain n-3 polyunsaturated fatty acid; LD, lipid droplet; LPCAT3, lysophosphatidylcholine acyltransferase 3; PL, phospholipid; PUFA, polyunsaturated fatty acid; SDA, stearidonic acid; TAG, triacylglycerol.

The five ALA fates described below differ in their generality across tumor contexts. Membrane phospholipid incorporation and fatty acid β-oxidation represent common cross-cutting fates: they occur to varying degrees in virtually all tumor cells that take up ALA and do not in themselves define a distinct tumor phenotype. By contrast, FADS/ELOVL-mediated conversion, lipid-droplet storage, and peroxidation-prone phospholipid enrichment are state-dependent fates: each becomes dominant only under specific molecular conditions and corresponds to one of the three tumor metabolic states described in Section 6. This distinction explains why five fates give rise to three, not five, dominant tumor metabolic states. **Figure 2** illustrates the biochemical routing of ALA through these five competing fates and their principal biological outputs, whereas **Table 1** summarizes the corresponding molecular regulators and interpretations.

**Table 1:** Five ALA fate routes and their relationship to tumor biological outputs.

ALA fate	Key molecular regulators	Predicted biological output	Interpretation
Membrane phospholipid incorporation	FATP/FABP/ACSL activity; phospholipid remodeling; omega-6 competition	Altered membrane unsaturation, raft composition, receptor localization, and peroxidation susceptibility	Cross-cutting route that may remodel signaling platforms and modify multiple phenotypes
FADS/ELOVL-mediated conversion	FADS2, FADS1, ELOVL5, ELOVL2; genotype; substrate availability	Generation of EPA, DPA, DHA, and related PUFA species; altered lipid mediator balance	Dominant in conversion-prone states; ALA behaves as a precursor only when conversion capacity is sufficient
Lipid-droplet storage and buffering	DGAT1/2; neutral lipid synthesis; lipid-droplet capacity; oxidative stress adaptation	Reduced exposure of PUFA acyl chains to peroxidation; metabolic lipid reserve	Dominant in storage-buffered states; may attenuate ALA-induced oxidative vulnerability
Fatty acid β-oxidation (FAO) and energy use	Nutrient stress; adipocyte-rich niche; mitochondrial FAO capacity; signalingAMP-activated protein kinase (AMPK)/mechanistic target of rapamycin (mTOR) signaling	ATP production, redox cofactor generation, and survival under stress	Cross-cutting adaptive route; may reduce signaling or peroxidation-active ALA pools
Peroxidation-prone phospholipid enrichment	ACSL4, LPCAT3, GPX4, FSP1, GSH status	Lipid peroxide accumulation and ferroptosis-like death when antioxidant defense is insufficient	Dominant in peroxidation-vulnerable states; attractive but incompletely validated for ALA specifically

Many malignant cells depend on external fatty acids in addition to *de novo* synthesis (36). Spatial single-cell isotope tracing has revealed substantial heterogeneity in the relative contribution of fatty-acid uptake versus *de novo* synthesis across cancer cells, indicating that reliance on exogenous lipids can vary markedly within tumors signaling (37). Lipid-rich niches further amplify this process: cluster of differentiation 36 (CD36)-positive metastasis-initiating cells respond to dietary lipids (38), adipocyte-derived lipids support melanoma progression through fatty acid transport protein (FATP) family members (39), and adipocytes promote ovarian cancer metastasis by providing fatty acids for rapid tumor growth (40). These findings are extrapolated from non-ALA lipid systems to establish a general principle: tumor cells actively scavenge and route exogenous fatty acids in a niche-dependent manner (41); whether ALA follows the same routing priorities in these specific contexts requires direct testing with ALA.

ALA is distinctive because it enters cells as a preformed polyunsaturated substrate. Unlike saturated or monounsaturated fatty acids, ALA carries three double bonds and immediately alters the unsaturation landscape available for membrane remodeling or lipid peroxidation. Direct studies in breast cancer cells show that ALA can be incorporated into phosphoglycerides in MCF-7 cells and can suppress growth in selected models, including MCF-7 and human epidermal growth factor receptor 2 (HER2)-overexpressing BT-474 cells (42–44). The key implication is that ALA uptake is not biologically neutral. Once imported and activated, ALA must therefore be allocated among the five competing intracellular fates discussed below.

### 4.1 Membrane phospholipid incorporation

A major fate of ALA is incorporation into membrane phospholipids, which links ALA availability to receptor biology and signaling platform organization. This incorporation may alter acyl-chain composition, membrane packing, lipid-raft organization, and the spatial distribution of signaling receptors and adhesion molecules (32). Direct ALA-specific raft studies in cancer remain limited, but ALA incorporation into phospholipids has been documented in breast cancer cells (42). The mechanistic consequences of this incorporation are extrapolated from EPA and DHA studies: EPA and DHA incorporation into lipid rafts reduces raft cholesterol and sphingomyelin, decreases epidermal growth factor receptor (EGFR) and C-X-C chemokine receptor type 4 (CXCR4) localization, and alters downstream signaling relevant to apoptosis and migration (45, 46); whether 18:3n-3-containing phospholipids produce comparable raft remodeling requires direct testing with ALA.

This membrane perspective has an important conceptual implication: ALA does not need to bind a single oncogenic target to be biologically active. By modifying the lipid environment surrounding receptors and signaling complexes, ALA-related phospholipid remodeling may alter HER2, EGFR, CXCR4, phosphoinositide 3-kinase/protein kinase B (PI3K/AKT), mitogen-activated protein kinase (MAPK), and inflammatory signaling in a subtype-dependent manner — though this inference is mechanistically plausible but not ALA-specific, as the cited evidence derives from longer-chain n-3 PUFA systems (46). This also explains why different studies report different signaling outcomes: the membrane effect of ALA depends on baseline phospholipid composition, cholesterol content, desaturase capacity, and competing fatty acids (43).

### 4.2 FADS/ELOVL-mediated conversion

The FADS/ELOVL axis is central to ALA fate, and FADS2 functions as a primary switch gate. If FADS2-dependent entry into the conversion pathway is inefficient, ALA may accumulate as 18:3-containing lipids or be diverted toward oxidation or storage. If FADS/ELOVL activity is high, ALA-derived intermediates may progress toward EPA, docosapentaenoic acid, DHA, or related long-chain PUFA species with distinct signaling and redox properties (29). The biological consequence is therefore not determined by ALA availability alone, but by the enzymatic state of the cell.

Evidence from mammary models illustrates this bottleneck. MCF-7 breast cancer cells incorporated ALA into phosphoglycerides but showed limited production of EPA or DHA compared with non-malignant mammary epithelial cells (43). In MDA-MB-231 cells, stearidonic acid generated downstream n-3 products more efficiently than ALA, supporting the idea that the initial FADS2-dependent desaturation step can constrain ALA processing (47). These findings argue against a linear model in which ALA is automatically converted to long-chain omega-3 PUFAs.

The expression of FADS2 and its downstream partners is not uniform across tumor types, and this heterogeneity is the molecular basis for the conversion-prone state described in Section 6.1. In triple-negative breast cancer (TNBC), high FADS1/2 expression is associated with active PUFA biosynthesis and poor prognosis, and is regulated at the epigenetic level (48). In gastric cancer, an opposite pattern is observed: intestinal-type tumors silence FADS1 and ELOVL5 through promoter DNA methylation, rendering them unable to generate arachidonic acid from linoleic acid and consequently resistant to ferroptosis (49). This tumor-type-specific epigenetic silencing versus activation of the FADS/ELOVL axis represents the mechanistic basis for the binary routing switch described in this section.

In breast cancer cell lines, FADS2 activity is substantially lower in malignant than in non-malignant mammary epithelial cells (43), suggesting that transformation itself may suppress conversion capacity in some contexts — though whether this reflects promoter methylation, transcription factor loss, or substrate competition remains unclear. At the germline level, FADS1/2 polymorphisms (rs174537, rs498793) further modify desaturase efficiency and have been associated with differential prostatic ALA handling and tumor aggressiveness (50). Collectively, these data indicate that FADS2 activity in tumors is determined by a hierarchy of regulators — epigenetic state, transcriptional programs, and germline genotype — that operate independently of dietary ALA supply and must be characterized before ALA fate can be predicted in any given tumor context.

Elongases further influence whether ALA-derived intermediates become functionally relevant lipid species. ELOVL5 promotes proliferation and invasion in renal cell carcinoma and represents a potentially targetable fatty acid elongase in prostate cancer (51, 52). More broadly, PUFA biosynthesis shapes ferroptosis sensitivity in gastric cancer leukemia and triple-negative breast cancer, where FADS1/2 activity influences lipid composition and ferroptotic vulnerability (48, 49). Conversely, downregulation of ELOVL5 can promote breast cancer metastasis through lipid-droplet accumulation and transforming growth factor beta (TGF-β) receptor induction (53). These observations support a bidirectional fate-switch: altered elongase activity can route ALA-derived substrates toward conversion, storage, or redox-sensitive lipid pools depending on context.

### 4.3 Lipid-droplet storage and buffering

Lipid-droplet storage functions as a buffering fate that diverts ALA-derived acyl chains away from membrane phospholipids and peroxidation-prone lipid pools. Diacylglycerol acyltransferase 1/2 (DGAT1/2)-mediated esterification sequesters these acyl chains into neutral lipid reserves, thereby reducing their exposure to membrane oxidative environments (13). The extent of this buffering is influenced by lipid-droplet capacity, nutrient availability, and oxidative stress adaptation. Tumors with strong neutral-lipid storage capacity may therefore show weaker redox or ferroptosis-related responses to ALA despite efficient uptake.

### 4.4 Fatty acid β-oxidation and energy use

Fatty acid β-oxidation (FAO) represents a distinct substrate-consuming fate in which ALA-derived acyl chains are directed toward mitochondrial energy metabolism rather than structural or peroxidation-prone lipid pools. Under nutrient stress, this route may support adenosine triphosphate (ATP) production and redox cofactor generation. In colon cancer, adipocyte-derived fatty acids promote mitochondrial FAO and autophagy under nutrient deprivation (54), although this evidence is not ALA-specific. The framework therefore predicts that high FAO capacity may reduce the proportion of ALA available for membrane remodeling, conversion, or lipid peroxidation, but this possibility requires direct testing with labeled ALA.

### 4.5 Peroxidation-prone phospholipid enrichment

Acyl-CoA synthetase long-chain family member 4 (ACSL4) activates long-chain polyunsaturated fatty acids to form acyl-CoA species, whereas lysophosphatidylcholine acyltransferase 3 (LPCAT3) facilitates their incorporation into membrane phospholipids. Together, these enzymes can expand the peroxidation-prone phospholipid pool defense (55, 56). Whether they directly route unconverted ALA into ferroptosis-relevant phospholipids, or whether prior FADS/ELOVL-mediated conversion is required, remains incompletely defined. The outcome depends critically on glutathione peroxidase 4 (GPX4) and ferroptosis suppressor protein 1 (FSP1) defense capacity: when both are intact, PUFA-phospholipid enrichment is tolerated; when either is compromised, lipid peroxide accumulation can reach cytotoxic levels (57, 58). In ovarian cancer, combined stearoyl-CoA desaturase 1 (SCD1)/FADS2 blockade lowers GPX4 activity and the ratio of reduced glutathione (GSH) to oxidized glutathione (GSSG) and triggers ferroptotic death, illustrating how disruption of the lipid-antioxidant balance tips the system toward oxidative collapse (59). This conditional relationship — PUFA-phospholipid loading becomes lethal only when antioxidant defense is insufficient — defines the peroxidation-vulnerable state described in Section 6.3.

## 5 Biological outputs of altered ALA fate

The biological effects attributed to ALA should be interpreted as outputs of the fate-switch described in Section 4. The following subsections follow the fate-output mapping in **Table 1**: proliferation and apoptosis are discussed mainly as outputs of membrane and redox fates; inflammatory and receptor signaling as outputs of membrane incorporation and conversion; ferroptosis as a conditional output of peroxidation-prone phospholipid enrichment; and migration or metastasis as a context-dependent output of membrane, oxidative, and host-niche effects. Where ALA-specific evidence is limited, this review distinguishes direct ALA data from evidence extrapolated from broader PUFA biology.

### 5.1 Proliferation, survival, and apoptosis

A recurring experimental observation is that ALA can suppress tumor-cell growth in selected models. In estrogen-positive MCF-7 breast cancer cells, ALA reduced growth and promoted a pro-apoptotic shift (44). Similar growth-inhibitory effects have been reported in oral squamous cell carcinoma and osteosarcoma models (60, 61), and ALA has also been shown to inhibit proliferation in renal cell carcinoma cells via peroxisome proliferator-activated receptor gamma (PPAR-γ) activation (62). These findings are consistent with the membrane incorporation fate: ALA-enriched phospholipid pools may alter membrane unsaturation and increase peroxidation pressure beyond cellular buffering capacity — a mechanistic interpretation extrapolated from broader PUFA-phospholipid literature rather than demonstrated directly for ALA in these models.

Mechanistically, apoptosis is the best-documented endpoint and is most plausibly interpreted as a consequence of membrane and redox disruption. In MCF-7 cells, ALA increased BCL-2-associated X protein (BAX) expression, decreased B-cell lymphoma 2 protein (BCL-2) expression, promoted cytochrome c release, and induced poly(ADP-ribose) polymerase (PARP) cleavage (44). In oral squamous cell carcinoma, ALA activated c-Jun N-terminal kinase (JNK)/c-Jun signaling, increased Fas ligand (FasL), and engaged extrinsic and intrinsic apoptotic cascades (60). Osteosarcoma studies also reported endoplasmic-reticulum-stress-associated signaling after ALA treatment (61). These outcomes should not be generalized as universal ALA effects. They represent outputs that may emerge when membrane phospholipid incorporation increases lipid peroxide pressure faster than the tumor’s antioxidant systems can neutralize it.

### 5.2 Inflammatory and receptor signaling

ALA-related metabolism may alter inflammatory tone by competing with omega-6 substrates at the level of lipid mediator production and lipid-sensing receptors. In renal carcinoma cells, ALA inhibited proliferation while increasing PPAR-γ activity and reducing cyclooxygenase-2 (COX-2) expression (62). In breast cancer cells, ALA reduced prostaglandin E2 (PGE2) production compared with arachidonic-acid-rich conditions; however, the magnitude of this effect was greater for stearidonic acid, which undergoes more efficient downstream conversion to long-chain n-3 intermediates than ALA (63), suggesting that the anti-inflammatory potency of ALA may depend substantially on its conversion efficiency rather than on 18:3n-3 incorporation alone — a relationship that requires direct testing with ALA under controlled conversion conditions.

Receptor signaling shows similar context dependence and is best understood through the membrane fate lens. Evidence that n-3 PUFA incorporation alters lipid raft composition and reduces EGFR and CXCR4 localization derives from EPA and DHA studies (46, 64) and is mechanistically plausible but not ALA-specific; whether 18:3n-3 phospholipids produce equivalent raft displacement at physiologically relevant incorporation levels remains untested. ALA may contribute to this process directly through 18:3n-3 phospholipid incorporation or indirectly through conversion to longer-chain omega-3 species (63). The signaling consequences are not uniformly inhibitory. They depend on membrane composition, receptor distribution, and downstream metabolic conversion, all of which vary by tumor subtype.

### 5.3 Ferroptosis susceptibility

The relationship between ALA and ferroptosis requires careful qualification on two levels: structural and contextual.

Structurally, ALA (18:3n-3) carries fewer bis-allylic C–H positions than longer-chain PUFAs such as arachidonic acid (AA, 20:4) or DHA (22:6), which are the primary sites of lipid radical propagation (65). ALA therefore has an intrinsically lower peroxidation rate than these species when incorporated directly into membrane phospholipids. Its capacity to sensitize tumor cells to ferroptosis is thus expected to depend on whether it is first elongated toward longer-chain peroxidation-competent PUFA via FADS/ELOVL activity, or whether it exerts pressure as 18:3-containing phospholipids alone (49, 66).

Direct ALA-specific evidence in cancer is limited but instructive. In pancreatic cancer cell lines, ALA induced lipid peroxidation and suppressed tumor growth in a ferrostatin-1-reversible manner, satisfying a standard mechanistic criterion for ferroptosis (67). However, the same study found that AA and EPA produced substantially stronger ferroptotic responses than ALA, and that ALA failed to induce ferroptosis in colorectal adenocarcinoma cells — illustrating cancer-type specificity that is consistent with the fate-switch framework (67). Notably, in non-tumor models, ALA activated the nuclear factor erythroid 2-related factor 2 (Nrf2)/solute carrier family 7 member 11 (SLC7A11)/GPX4 axis and suppressed ferroptosis (68), indicating that ALA’s interaction with ferroptosis machinery is bidirectional and determined by the antioxidant defense state of the receiving cell. The broader PUFA–ferroptosis literature provides the mechanistic context for these observations, but its applicability to ALA remains dependent on tumor-specific conversion and phospholipid-routing capacity (48, 49, 55).

ALA is therefore positioned here as a conditional ferroptosis-relevant substrate rather than a proven universal sensitizer. Tumors with active FADS/ELOVL conversion, high ACSL4/LPCAT3 activity, and weak GPX4/FSP1 defense represent the most plausible candidates for ALA-driven ferroptotic sensitization (57, 58). Validation requires lipidomics-based fate tracing and ferrostatin-1 rescue experiments rather than inference from gene expression or non-ALA PUFA data alone.

### 5.4 Migration, invasion, and metastasis

ALA may influence metastatic traits through mechanisms that converge on membrane incorporation and redox fate. The mechanistic basis for this influence — that altered membrane unsaturation affects focal adhesion dynamics, integrin clustering, and matrix-remodeling programs — is extrapolated from PUFA-phospholipid remodeling studies and has not been demonstrated with ALA specifically (12). In oral squamous cell carcinoma, lower ALA concentrations suppressed migration and invasion, accompanied by reduced expression of the epithelial–mesenchymal transition (EMT)-associated transcription factor Twist and matrix metalloproteinase (MMP)-2 and MMP-9 proteins (60). In osteosarcoma cells, ALA suppressed invasive behavior (61). These observations are consistent with ALA-related phospholipid remodeling weakening EMT-like programs program and cell-matrix interaction, but the upstream mechanism linking ALA incorporation to these endpoints requires direct testing with ALA rather than inference from broader PUFA remodeling data.

At the same time, metastasis is especially sensitive to host-niche context. Evidence that the lipid metabolic environment of metastatic niches alters oxidative defense — including the observation that lymph fluid protects melanoma cells from ferroptosis (69) — is mechanistically plausible as a framework for ALA but derives from non-ALA systems and requires direct testing with ALA in metastatic niche models. The fate-switch framework therefore predicts that anti-metastatic effects should not be treated as intrinsic properties of ALA. They should be tested in relation to where ALA-derived lipids accumulate, whether oxidative pressure is imposed on tumor cells or stromal compartments, and whether the metastatic niche buffers or amplifies lipid peroxidation (13).

## 6 Predictive stratification framework: three dominant tumor metabolic states

The fate-switch framework implies that future ALA studies should move beyond the binary question of benefit versus harm. A more useful question is which tumors are likely to convert ALA exposure into biologically meaningful lipid remodeling, and in which direction. Although Section 4 describes five possible ALA fates, the current evidence is most operationally organized around three dominant tumor metabolic states: conversion-prone, storage-buffered, and peroxidation-vulnerable. Membrane remodeling and fatty acid β-oxidation (FAO) are retained as cross-cutting fate routes that can modify these states rather than as fully independent phenotypes at this stage.

These states should not be interpreted as mutually exclusive categories. They represent dominant positions along a continuum of lipid routing. A tumor may be both conversion-prone and peroxidation-vulnerable if FADS2/FADS1/ELOVL activity and ACSL4-driven phospholipid incorporation are high while GPX4/FSP1 defense is weak (48, 55). Conversely, a tumor may shift toward a storage-buffered state under nutrient stress, therapy, or adipocyte-derived lipid pressure (54). Primary tumors and metastases may also differ in their dominant state, as suggested by evidence that the lipid metabolic environment of metastatic niches can substantially alter PUFA handling and oxidative defense relative to the primary tumor (69). The value of the framework is therefore not to force each tumor into a rigid group, but to define testable routing hypotheses before ALA exposure.

### 6.1 Conversion-prone state

Tumors in a conversion-prone state would express sufficient FADS2, FADS1, ELOVL5, and ELOVL2 activity to convert ALA into longer-chain omega-3 PUFA species. Indirect support for this concept comes from studies showing that FADS1/2-high TNBC subsets exhibit active PUFA biosynthesis and heightened ferroptosis susceptibility (48), and that ELOVL5/FADS1 activity drives conversion of exogenous linoleic acid to arachidonic acid in mesenchymal-type gastric cancer cells (49). These findings establish that the FADS/ELOVL axis can function as an active conversion route in specific tumor contexts, but they do not demonstrate ALA-specific routing: both studies used omega-6 substrates or focused on endogenous PUFA synthesis rather than exogenous ALA. Whether the same enzymatic capacity efficiently processes ALA as an omega-3 substrate in these or similar models remains to be established by isotope-tracing experiments with labeled ALA.

A testable hypothesis is that FADS2-high, ACSL4-high, GPX4-low tumor models would be more likely to show ALA-driven lipid peroxide accumulation and sensitization to ferroptosis inducers than FADS2-low or ACSL4-low models (70). The PUFA biosynthesis data from gastric cancer and TNBC suggest which tumor contexts are worth prioritizing for such tests (48, 49), but direct confirmation requires isotope-tracing with labeled ALA, lipidomics of ALA-derived phospholipid species, and ferrostatin-1 rescue experiments rather than inference from FADS expression or omega-6 substrate data alone.

Based on the established roles of FADS2 and downstream FADS/ELOVL enzymes in the conversion of ALA to long-chain n-3 PUFAs, as well as the variable ALA-processing capacity observed across mammary epithelial and breast cancer cell models, we hypothesize that FADS/ELOVL-high tumor models will convert a greater proportion of exogenous ALA into long-chain n-3 PUFA species than FADS/ELOVL-low models (25, 29, 43, 47). This prediction should be tested by stable-isotope tracing with labeled ALA and lipidomic quantification of ALA-derived EPA, docosapentaenoic acid (DPA), DHA, and their phospholipid species. Genetic or pharmacological inhibition of FADS2 would provide additional validation if it reduces the formation of these downstream products.

A secondary prediction applies when the conversion-prone state overlaps with a peroxidation-vulnerable phenotype. Based on broader PUFA evidence, tumors with active FADS/ELOVL conversion, high ACSL4 and/or LPCAT3 activity, and limited GPX4/FSP1 defense may preferentially incorporate ALA-derived long-chain PUFAs into peroxidation-prone phospholipids and thereby exhibit increased ferroptotic vulnerability (48, 49, 55–58). This overlapping phenotype should be evaluated using oxidized-phospholipid lipidomics, lipid-peroxidation assays, and rescue experiments with ferrostatin-1 or vitamin E.

### 6.2 Storage-buffered state

Tumors in a storage-buffered state preferentially channel exogenous fatty acids, including PUFAs, into lipid droplets rather than membrane phospholipids or oxidative pathways. Lipid-accumulating tumors and tumors in adipocyte-rich niches may show this state under lipid-abundant conditions (13). In this setting, ALA taken up from the microenvironment may be esterified into neutral lipid pools, reducing its exposure to oxidative enzymes and membrane environments. The predicted response is weak or absent anti-proliferative, apoptotic, or ferroptosis-related effects because polyunsaturated acyl chains are sequestered away from peroxidation-prone membranes.

A testable hypothesis is that inhibiting DGAT-dependent neutral-lipid storage before ALA exposure may redirect a greater proportion of ALA-derived acyl chains from lipid droplets toward membrane phospholipids and increase oxidative vulnerability, as suggested by broader PUFA evidence showing that DGAT inhibition redistributes PUFAs from triacylglycerols to phospholipids and restores ferroptosis sensitivity (71). This hypothesis would be supported if DGAT inhibition increased ALA-containing phospholipids, lipid-peroxide accumulation, and redox-sensitive cell death, and would be weakened if these changes were absent despite effective suppression of lipid-droplet formation.

### 6.3 Peroxidation-vulnerable state

Tumors in a peroxidation-vulnerable state have high PUFA-phospholipid enrichment, high ACSL4 and/or LPCAT3 activity, weak GPX4/FSP1/glutathione defenses, or some combination of these features (55, 56). These tumors may be closer to the ferroptosis threshold and may be more sensitive to additional PUFA loading. In this state, ALA exposure, even without complete conversion to long-chain PUFAs, could increase peroxidizable 18:3-containing phospholipids and lower the threshold for oxidative collapse.

A testable hypothesis is that tumors with a high ACSL4/GPX4 or ACSL4/FSP1 balance would be more likely to respond to ALA with lipid peroxide accumulation and growth suppression than tumors with low ACSL4 or strong peroxide defense. This prediction should be tested with functional rescue experiments, including ferrostatin-1, vitamin E, GPX4 modulation, or glutathione restoration (72). Without rescue experiments, lipid peroxidation cannot be confidently linked to ALA-driven ferroptotic vulnerability.

### 6.4 Operational assignment and falsifiability

Operationally, these states should be assigned using a combination of transcriptomic or proteomic markers and functional lipid assays rather than single-gene expression alone. A conversion-prone state would require high FADS/ELOVL expression together with detectable ALA-derived long-chain PUFA species by lipidomics or isotope tracing (49). A storage-buffered state would require lipid-droplet accumulation and preferential incorporation of ALA into neutral lipids. A peroxidation-vulnerable state would require PUFA-phospholipid enrichment, high ACSL4/LPCAT3 activity, and limited GPX4/FSP1/glutathione defense (55, 73).

The framework is intentionally falsifiable. If FADS2-high, ACSL4-high, GPX4-low models do not accumulate ALA-derived peroxidizable lipids or show redox-sensitive growth effects, the conversion-peroxidation prediction would require revision (70). If storage-buffered models respond strongly to ALA despite neutral lipid sequestration, the buffering hypothesis would be weakened. If ACSL4/GPX4 status fails to correlate with ALA-induced lipid peroxidation across matched models, the peroxidation-vulnerable state would need refinement (55). These negative outcomes would be informative because they would identify which routing assumptions are wrong rather than simply concluding that ALA is ineffective. The operational criteria and falsification tests for the proposed ALA responsiveness states are summarized in **Table 2**.

**Table 2:** Operational criteria and falsification tests for ALA responsiveness states.

Dominant state	Operational criteria	Expected ALA response	Recommended validation approach	Falsification test
Conversion-prone	High FADS2/FADS1/ELOVL5/ELOVL2 plus detectable ALA-derived long-chain PUFA by lipidomics or tracing	Greater formation of ALA-derived long-chain n-3 PUFA species, with downstream lipid-mediator or redox effects depending on phospholipid routing and antioxidant-defense capacity	Labeled-ALA tracing; lipidomics of ALA-derived long-chain PUFAs; FADS2 inhibition or knockdown	FADS/ELOVL-high models fail to form ALA-derived long-chain PUFA species after labeled ALA exposure
Storage-buffered	Lipid-droplet accumulation; DGAT activity; preferential routing of ALA into neutral lipids	Weak ALA-induced apoptosis or ferroptosis because ALA is sequestered away from membranes	Neutral-lipid and phospholipid fractionation; lipid-droplet imaging; DGAT inhibition	Storage blockade does not increase ALA-containing phospholipids, lipid peroxides, or growth effects
Peroxidation-vulnerable	High PUFA-phospholipid enrichment; high ACSL4 and/or LPCAT3 activity; low GPX4/FSP1/GSH defense	Greater likelihood of redox-sensitive growth suppression or ferroptosis sensitization after ALA exposure	Oxidized-phospholipid lipidomics; C11-BODIPY; ferrostatin-1 or vitamin E rescue; GPX4 modulation	ACSL4/GPX4 status does not predict ALA-induced lipid peroxidation across matched models

These states represent dominant predicted phenotypes rather than mutually exclusive categories. The magnitude and direction of ALA responses may be modified by ω-6 competition, host metabolic context, peroxide-defense capacity, redox state, FADS genotype, and sex.

For practical translational research, a minimal candidate panel could include FADS2 as an indicator of conversion capacity, DGAT1/2 or lipid-droplet abundance as indicators of storage capacity, ACSL4 as an indicator of PUFA activation, and GPX4 together with FSP1 as indicators of peroxide-defense capacity. These markers can be assessed using immunohistochemistry, transcriptomics, or proteomics, but none can establish ALA fate alone. Functional confirmation should therefore include labeled-ALA tracing or lipidomics, together with ferrostatin-1 or vitamin E rescue for redox-dependent effects and DGAT perturbation for storage-buffered states.

## 7 Nutritional and therapeutic implications

### 7.1 ALA as a candidate modifier, not a stand-alone anticancer intervention

ALA is accessible through common plant foods and therefore represents a practical nutritional variable for cancer research (20). However, current evidence does not justify portraying ALA as a universal anticancer nutrient, and the epidemiological record illustrates precisely the heterogeneity that the fate-switch framework is designed to explain.

At the population level, a large-scale dose-response meta-analysis of 41 prospective cohort studies (n = 1,197,564) found that higher dietary ALA intake was associated with reduced all-cause and cardiovascular mortality but with a slightly elevated cancer mortality signal that did not reach consistency across subgroup analyses and may reflect residual confounding rather than a direct effect of ALA, while higher circulating ALA biomarker levels showed a pattern distinct from dietary intake — a divergence that itself reflects the conversion and routing problem central to this review (74). In colorectal cancer specifically, a meta-analysis of 15 cohorts (861,725 participants, 12,239 cases) found no association between dietary ALA intake and colorectal cancer risk (summary relative risk: 1.03), yet blood ALA biomarker levels were associated with a 10% reduction in risk per 0.1% increment (17). The systematic divergence between dietary intake and circulating biomarkers across these large datasets is precisely what the fate-switch framework predicts: intake does not equal fate, and the same dietary ALA exposure produces different tissue-level outcomes depending on conversion efficiency, storage capacity, and competing omega-6 background.

The prostate cancer literature further illustrates this problem. While dietary ALA intake shows weak or inconsistent associations with prostate cancer risk across cohorts, prostatic tissue ALA concentration — independent of intake — has been positively associated with markers of tumor aggressiveness, and FADS-related single-nucleotide polymorphisms (SNPs) (particularly rs174537 and rs498793) modify this relationship (50). This suggests that the same dietary ALA may behave differently depending on individual desaturase genotype, which current epidemiological studies rarely capture.

Food-based interpretation also requires caution: flaxseed, walnuts, soybean oil, and canola oil differ in accompanying nutrients, omega-6 content, fiber, phytochemicals, and lipid-delivery form (20). Effects of ALA-rich foods cannot be attributed exclusively to ALA without measuring tissue lipid incorporation and downstream metabolic fate. Human intervention studies indicate that circulating ALA exposure varies according to the food matrix and background fatty-acid composition (29, 75). However, controlled data directly quantifying ALA incorporation into human tumor tissue under different dietary conditions remain scarce. Future nutritional studies should therefore combine dietary assessment with plasma lipid profiling and, where feasible, tumor-tissue lipidomics. Therefore, ALA should be treated as a candidate modifier of tumor lipid metabolism rather than as a stand-alone anticancer intervention, and future epidemiological analyses should stratify by tissue ALA biomarker, FADS genotype, and cancer molecular subtype rather than relying on dietary intake alone.

### 7.2 Combination strategies should be phenotype-matched and hypothesis-driven

ALA may be relevant as a metabolism-modifying adjuvant hypothesis, but only if matched to tumor lipid phenotype. For peroxidation-vulnerable states, combining ALA with GPX4 inhibition, glutathione-depleting approaches, or ferroptosis-oriented strategies is mechanistically plausible and should be prioritized for experimental testing (76, 77). For conversion-prone states, the relevant question is whether ALA-derived long-chain PUFA species actually enter ACSL4-dependent phospholipids (55). For storage-buffered states, ALA alone would be expected to have weaker effects, and the more rational experimental strategy would be to disrupt lipid-droplet buffering before PUFA loading (70, 71).

These strategies remain experimental hypotheses rather than clinically actionable recommendations. In tumors with strong lipid-droplet storage capacity or robust GPX4/FSP1 defense, ALA supplementation may be biologically weak, neutral, or unpredictable regardless of dose (73). Phenotype-matched design is therefore not a refinement but a prerequisite for interpretable ALA studies.

### 7.3 Requirements for future translation

Several problems must be solved before ALA can be translated into oncology nutrition or adjuvant strategies. First, exposure needs standardization. *In vitro* studies often apply free ALA at defined concentrations, while *in vivo* exposure depends on digestion, absorption, lipoprotein transport, tissue uptake, and competition with other fatty acids. Flaxseed oil, milled flaxseed, whole flaxseed, and purified ALA may produce different plasma and tissue ALA profiles and should not be treated as interchangeable (75).

Second, future studies should measure fate rather than intake alone. Lipidomics can identify ALA-containing phospholipids, neutral lipids, and downstream PUFA species in tumor and stromal compartments. Stable-isotope tracing with labeled ALA can determine whether ALA is converted, stored, oxidized, or incorporated into membranes. Redox assays can assess lipid peroxidation burden, GPX4 dependence, glutathione status, and ferroptosis markers (76). Spatial and single-cell approaches may reveal whether ALA handling differs between tumor cells, cancer-associated fibroblasts, immune cells, and adipocyte-rich niches (37).

Third, clinical research should be stratified according to the framework described in Section 6. Patient selection and experimental models should consider tumor type, molecular subtype, FADS2/ELOVL5 expression, ACSL4/GPX4/FSP1 status, lipid-droplet capacity, omega-6 dietary background, obesity, insulin resistance, and desaturase-related genotype. Sex should also be included as an explicit stratification variable: as noted in Section 3, women exhibit higher ALA-to-EPA/DHA conversion efficiency than men due to estrogen-regulated desaturase activity, which means that the same ALA exposure may produce different tissue lipid profiles and different biological outputs across sexes (33). FADS1/2 genetic polymorphisms — particularly rs174537, rs174547, and rs498793, which are known to modify desaturase efficiency and have been associated with differential PUFA metabolism in breast and prostate cancer — should be captured prospectively rather than treated as uncontrolled confounders (78). Without these variables, studies may continue to pool tumors from different metabolic states and generate mixed results that obscure real effects and null effects alike.

Breast cancer represents a logical initial setting for framework validation because direct studies have demonstrated ALA incorporation, metabolic processing, and growth modulation in breast cancer models, while subtype-specific FADS1/2 activity provides an additional basis for metabolic stratification (42–44, 48). Pancreatic cancer is another priority because ALA-induced lipid peroxidation and ferrostatin-1-reversible growth suppression have been reported (67). Gastric and ovarian cancers provide additional mechanistic models for testing conversion and redox-state hypotheses, although the available evidence in these tumors is currently derived mainly from non-ALA PUFA systems (49, 59). These cancer types should therefore be regarded as priority experimental models rather than established clinical indications for ALA intervention.

### 7.4 Potential risks and unintended consequences of ALA loading

The risks of high-dose ALA have received little attention in the cancer-focused literature and must be acknowledged before translational applications are considered. Three areas warrant caution. First, high n-3 PUFA loading can increase membrane phospholipid peroxidation in normal tissues in a dose-dependent manner (79), and recent evidence demonstrates that DHA induces ferroptotic death in normal cells under conditions of insufficient antioxidant defense (80); whether ALA produces comparable pro-oxidant effects in non-tumor tissues at supplementation-relevant doses has not been directly examined. Second, high-dose n-3 PUFAs suppress CD4+ T cell activation, dendritic cell maturation, and natural killer (NK) cell function via lipid-raft remodeling (81, 82); whether ALA contributes to this immunosuppressive effect directly or through partial conversion is mechanistically plausible but not ALA-specific and requires direct testing. Third, n-3 PUFA enrichment of tumor membranes may sensitize cancer cells to anthracycline-induced oxidative stress (83), but whether this enrichment equally increases oxidative susceptibility of normal tissues — particularly the heart — remains unresolved (83). Similarly, although n-3 PUFA enrichment may enhance tumor-cell radiosensitivity through lipid peroxidation, its effects on ALA-enriched normal tissues remain insufficiently defined (84). Future studies involving ALA loading should therefore include normal tissue peroxidation markers, immune profiling, and treatment interaction endpoints alongside tumor-focused outcomes.

## 8 Conclusions and future perspectives

ALA should not be interpreted only as a dietary precursor of EPA and DHA. In cancer, its biological significance is likely to depend on tumor-specific lipid uptake, conversion, storage, oxidation, and phospholipid remodeling (8, 10–13). The same ALA exposure may therefore generate different biological outputs depending on the molecular and lipid-metabolic state of the receiving tumor.

The predictive fate-switch framework proposed here advances prior descriptions in three ways. First, it reframes ALA from a nutrient with inherent anticancer or protumor properties to a substrate whose output is determined by tumor lipid-handling machinery. Second, it identifies five competing fate routes and their molecular regulators, providing mechanistic specificity for experimental design (32). Third, it organizes tumor heterogeneity into three dominant but overlapping metabolic states - conversion-prone, storage-buffered, and peroxidation-vulnerable - each with distinct expected ALA responses (48, 49).

These predictions are testable and should be framed as hypotheses requiring direct validation. Across all three states, the common prediction is that ALA’s biological output is determined by intracellular routing rather than by exposure level. Conversion-prone tumors with high FADS/ELOVL and ACSL4 activity represent the most tractable starting point for ALA-ferroptosis studies (48, 55); storage-buffered tumors call for combination approaches that first disrupt neutral lipid sequestration (71); and peroxidation-vulnerable tumors may respond to ALA loading without prior conversion, provided antioxidant defenses are insufficient (76). The unifying experimental requirement is lipidomics-based fate confirmation before any mechanistic interpretation is drawn. Testing these predictions with lipidomics, isotope tracing, and rescue experiments would either support the framework or require its revision.

The practical implication is that future ALA research in oncology should be phenotype-first. Rather than asking whether ALA is beneficial or harmful across all cancers, studies should ask which tumor lipid-metabolic context converts ALA exposure into a functionally relevant fate. This approach may make ALA research more experimentally actionable and translationally informative while avoiding overstatement of ALA as a generalized anticancer nutrient.

The framework carries important limitations that define its appropriate scope. It applies only to tumor cells that actively take up and metabolize ALA; tumors with minimal exogenous lipid dependence, poor vascular access, or very low FATP/CD36 expression represent contexts where fate-routing predictions may not hold. It assumes that lipid metabolic reprogramming is a dominant feature of the tumor being studied; for tumors primarily driven by glycolytic or non-lipid metabolic programs, ALA fate is unlikely to be a functionally decisive variable. The three dominant states are analytically distinct but biologically overlapping. Although the framework accommodates such overlap conceptually, it does not yet provide quantitative criteria for resolving mixed phenotypes, such as tumors combining high FADS2 activity with strong lipid-droplet buffering. These mixed states should be prioritized for future flux-based and multivariate modelling. Finally, and most importantly, the framework is a hypothesis-generating tool grounded primarily in mechanistic inference; it does not constitute clinical evidence for ALA supplementation and should not be used to justify nutritional recommendations outside of controlled experimental settings. Ultimately, integrating tumor lipid phenotype with host dietary context may provide a rational foundation for personalized nutritional oncology, while remaining contingent on prospective experimental and clinical validation.

## Data Availability

The original contributions presented in the study are included in the article/supplementary material. Further inquiries can be directed to the corresponding author/s.
